# Enhanced excitability of small dorsal root ganglion neurons in rats with bone cancer pain

**DOI:** 10.1186/1744-8069-8-24

**Published:** 2012-04-03

**Authors:** Qin Zheng, Dong Fang, Jie Cai, You Wan, Ji-Sheng Han, Guo-Gang Xing

**Affiliations:** 1 Neuroscience Research Institute and Department of Neurobiology, Peking University, 38 Xue-Yuan Road, Beijing 100191, People's Republic of China; 2 Key Laboratory for Neuroscience of the Ministry of Education and the Ministry of Public Health, 38 Xue-Yuan Road, Beijing 100191, People's Republic of China

**Keywords:** Bone cancer pain, Hyperalgesia, hyperexcitability, Peripheral sensitization, Dorsal root ganglion, rat

## Abstract

**Background:**

Primary and metastatic cancers that affect bone are frequently associated with severe and intractable pain. The mechanisms underlying the development of bone cancer pain are largely unknown. The aim of this study was to determine whether enhanced excitability of primary sensory neurons contributed to peripheral sensitization and tumor-induced hyperalgesia during cancer condition. In this study, using techniques of whole-cell patch-clamp recording associated with immunofluorescent staining, single-cell reverse-transcriptase PCR and behavioral test, we investigated whether the intrinsic membrane properties and the excitability of small-sized dorsal root ganglion (DRG) neurons altered in a rat model of bone cancer pain, and whether suppression of DRG neurons activity inhibited the bone cancer-induced pain.

**Results:**

Our present study showed that implantation of MRMT-1 tumor cells into the tibial canal in rats produced significant mechanical and thermal hyperalgesia in the ipsilateral hind paw. Moreover, implantation of tumor cells provoked spontaneous discharges and tonic excitatory discharges evoked by a depolarizing current pulse in small-sized DRG neurons. In line with these findings, alterations in intrinsic membrane properties that reflect the enhanced neuronal excitability were observed in small DRG neurons in bone cancer rats, of which including: 1) depolarized resting membrane potential (RMP); 2) decreased input resistance (R_in_); 3) a marked reduction in current threshold (CT) and voltage threshold (TP) of action potential (AP); 4) a dramatic decrease in amplitude, overshot, and duration of evoked action potentials as well as in amplitude and duration of afterhyperpolarization (AHP); and 5) a significant increase in the firing frequency of evoked action potentials. Here, the decreased AP threshold and increased firing frequency of evoked action potentials implicate the occurrence of hyperexcitability in small-sized DRG neurons in bone cancer rats. In addiotion, immunofluorescent staining and single-cell reverse-transcriptase PCR revealed that in isolated small DRG neurons, most neurons were IB4-positive, or expressed TRPV1 or CGRP, indicating that most recorded small DRG neurons were nociceptive neurons. Finally, using in vivo behavioral test, we found that blockade of DRG neurons activity by TTX inhibited the tumor-evoked mechanical allodynia and thermal hyperalgesia in bone cancer rats, implicating that the enhanced excitability of primary sensory neurons underlied the development of bone cancer pain.

**Conclusions:**

Our present results suggest that implantation of tumor cells into the tibial canal in rats induces an enhanced excitability of small-sized DRG neurons that is probably as results of alterations in intrinsic electrogenic properties of these neurons. Therefore, alterations in intrinsic membrane properties associated with the hyperexcitability of primary sensory neurons likely contribute to the peripheral sensitization and tumor-induced hyperalgesia under cancer condition.

## Background

Bone cancer pain resulting from primary tumors or tumors that metastasize to bone is one of the most severe and intractable types of cancer pain, which reduces the quality of the cancer patient's life [1,2]. Bone cancer pain is generally consists of ongoing pain and breakthrough or incident pain, which are characterized by pathological symptoms, such as spontaneous pain, hyperalgesia, and allodynia [3,4]. Studies using rodent models of bone cancer pain have revealed that tumor growth in these models concurrently evokes mechanical and thermal hyperalgesia [5-7], sensitization of nociceptive dorsal horn neurons [6], sensitization of primary afferent C nociceptors that innervate skin overlying the tumor [8-10], and development of injury to primary afferent nerve fibers that innervate the tumor-bearing bone, where the injury is accompanied by a stereotypic set of cellular and neurochemical changes in the dorsal root ganglia (DRG) [11]. It is now suggested that sensitization of primary afferent C nociceptors innervating tissue near the tumor likely contribute to the chronic pain and hyperalgesia accompanying the cancer condition [9,10]. However, alterations in the intrinsic electrogenic properties and the excitability of primary afferent DRG neurons in the condition of bone cancer pain still remain largely unknown.

Electrophysiological recordings from primary sensory neurons with transected peripheral axons indicate that the somata in the DRG can become hyperexcitable [12,13]. Damage to the peripheral nervous system, which produces behavioural signs of neuropathic pain, induces long-lasting hyperexcitability of sensory neurons in diverse species [14,15]. In mammals, injury affecting the axons or somata of sensory neurons having their somata in DRGs often causes hyperexcitability that may lead to spontaneous firing, neuropathic pain, and paresthesias [16,17]. Increases in excitability in all types of DRG sensory neurons within weeks of axotomy have been found, although electrophysiological changes appear to vary among types of neurons and with the type of injury [13,18]. In large and medium A-cells axotomy induced by sciatic nerve section or spinal nerve ligation caused a reduction of excitation threshold, an increase in duration and in rise time of the action potential, whereas in small C-cells axotomy reduced action potential threshold but did not significantly change resting membrane potential, action potential duration, or maximal depolarization rate [12,13,18-20]. In addition, nerve injury led to a significant reduction of the rheobase, an index of neuronal excitability, in all types of cells [18,19]. It has been suggested that the nerve injury-induced increase in excitability is related to the depolarizing shift of the resting membrane potential (RMP) in C-cells and to the increase in apparent input resistance near the RMP in A-cells [18].

Enhanced excitability of nociceptive DRG neurons have also been observed in rodent models of inflammatory pain [21-23]. Decreases in durations of action potentials (C- and Aδ-fibre units) and afterhyperpolarisations (A-fibre units) occur in somata of nociceptive DRG neurons during hindlimb inflammation induced in young guinea-pigs by intradermal injections of complete Freund's adjuvant (CFA) into the ipsilateral leg and foot [21]. Hindlimb inflammation also causes a significant increase in conduction velocity of nociceptive primary sensory neurons during inflammation, as well as a significant decrease in the mean electrical threshold for eliciting the C and Aδ components of compound action potentials of both dorsal root and sural nerves [24]. The alterations in membrane properties of nociceptors likely permit higher discharge frequencies, and thus contributing to inflammatory hyperalgesia [22,24,25]. Inflammatory mediators, by increasing the excitability of DRG somata via augmentation of Nav1.9 current [26] or suppression of A-type K^+ ^current [23], may contribute to nociceptors' hyperexcitability during peripheral inflammation, or even contribute to chronic compression of the DRG (CCD)-induced neuronal hyperexcitability and to hyperalgesia and tactile allodynia [27].

In the present study, using whole-cell patch-clamp recording methods, we investigated whether the intrinsic electrogenic properties and the excitability of small-sized DRG neurons altered in a rat model of bone cancer pain. The aim of this study was to determine whether enhanced excitability of primary sensory neurons contributed to peripheral sensitization and tumor-induced hyperalgesia under cancer condition.

## Results

### Tumor-evoked mechanical allodynia and thermal hyperalgesia

**Figure 1:**
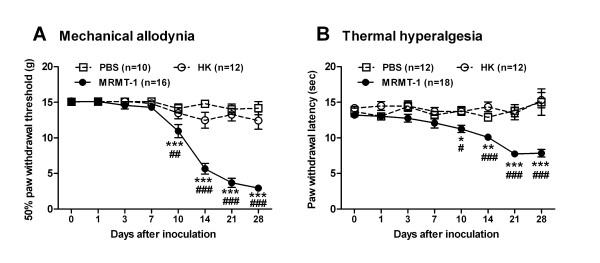
**Changes in 50% paw withdrawal threshold (PWT) to von Frey filaments and paw withdrawal latency (PWL) to radiant heat in rats with intra-tibial injections of MRMT-1 cells**. (**A**): 50% PWT; (**B**): PWL. Note that implantation of MRMT-1 tumor cells into the tibial canal in rats produced significant decreases in 50% PWT and PWL in contrast to PBS or heat-killed cells injection. *p < 0.05, **p < 0.01, ***p < 0.001, as compared with PBS-implanted rats; ^#^*p *< 0.05, ^##^*p *< 0.01, ^###^*p *< 0.001, as compared with heat-killed (HK) cells-implanted rats, two-way ANOVA followed by Bonferroni post-hoc test.

Consistent with previous reports [28,29], implantation of MRMT-1 tumor cells into the tibial canal in rats produced significant mechanical allodynia (Figure 1A). The 50% paw withdrawal threshold (PWT) to von Frey filaments in the tumor-bearing hind paw in MRMT-1 rats decreased from 15.08 ± 0.01 g of the baseline level to 10.95 ± 0.93 g by day 10 after implantation (*P *< 0.001). The further decrease in the 50% PWT was observed from day 14 (5.66 ± 0.75 g) to day 21 (3.68 ± 0.65 g) until the last testing session on day 28 (2.94 ± 0.35 g). In contrast, the 50% PWT of the injected hind paw in heat-killed (HK)- and PBS-implanted rats had no significant alteration during the time course of post-inoculation and were higher than those in MRMT-1 rats from day 10 after implantation (*P *< 0.001, *P *< 0.001).

Implantation of MRMT-1 tumor cells also produced thermal hyperalgesia (Figure 1B). Mean paw withdrawal latency (PWL) to the radiant heat stimulation was 13.20 ± 0.33 s prior to implantation of tumor cells. By day 10 after implantation, PWL began to decrease (11.26 ± 0.51 s, *P *< 0.05) in the tumor-bearing hind paw in MRMT-1 rats. The PWL decreased further to 7.85 ± 0.54 s by day 28 (*P *< 0.001). In contrast, the PWL of the injected hind paw in heat-killed (HK)- and PBS-implanted rats remained unchanged and were longer than those in MRMT-1 rats from day 10 after implantation (*P *< 0.001, *P *< 0.001). Thus, implantation of MRMT-1 tumor cells into the tibial canal in rats produced significant mechanical allodynia and thermal hyperalgesia, indicating the development of tumor-induced bone cancer pain.

### Tumor induced spontaneous discharges and tonic excitation in isolated small-sized DRG neurons

**Figure 2:**
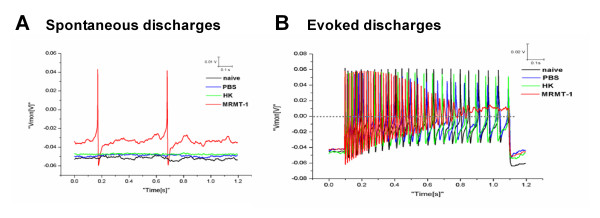
**Spontaneous discharges and evoked tonic discharges in small-sized DRG neurons in MRMT-1-rats**. (**A**): Representative of spontaneous discharges. (**B**): Representative of evoked discharges. The red trace represents the discharges recorded from representative small DRG neurons in MRMT-1-implanted rats. Note that implantation of MRMT-1 tumor cells into the tibial canal in rats provoked spontaneous discharges and evoked tonic discharges in MRMT-1-rats but not control rats.

To determine whether the excitability of primary sensory neurons increased in bone cancer rats, we examined the spontaneous activities and evoked discharges in small-sized DRG neurons in naïve, PBS-, HK-, and MRMT-1-rats. We found that implantation of MRMT-1 tumor cells into the tibial canal in rats provoked spontaneous discharges and tonic excitatory discharges evoked by 1-s, 300-pA depolarizing current pulse, recorded from small-sized DRG neurons. As shown in Figure 2A, in PBS (0/45)-, HK (0/49)-treated, or naïve (0/63) rats, no spontaneous activity was observed in all recorded small DRG neurons, whereas in MRMT-1-implanted rats, 9 of 58 (15.52%) recorded DRG neurons exhibited spontaneous discharges. Moreover, as compared with PBS (1/45, 2.22%)-, HK (1/49, 2.04%)-treated, or naïve (2/63, 3.17%) rats, more neurons (25/58, 43.10%) generated tonic excitatory discharges evoked by a depolarizing current pulse (1 s, 300 pA) in bone cancer rats (Figure 2B). The results suggested that enhanced excitability in small-sized DRG neurons occurred in bone cancer rats.

### Decreases in the depolarized current threshold (CT) and the membrane input resistance (R_in_) in small-sized DRG neurons in MRMT-1-rats

To investigate whether the tumor-induced enhancement of excitability in small-sized DRG neurons resulted from the alteration of intrinsic electrogenic properties, we measured the neuronal intrinsic membrane properties such as resting membrane potential (RMP), membrane input resistance (R_in_), and various parameters of evoked action potentials, which reflecting excitability (Table 1). In this study, the recorded small DRG neurons were initially divided into three types on the basis of the depolarized current threshold (CT) for eliciting the 1^st ^action potential: the low-firing threshold (LT) neurons (CT < 50 pA), the medium-firing threshold (MT) neurons (CT: 50 - 100 pA), and the high-firing threshold (HT) neurons (CT > 100 pA). The results showed that in contrast to control rats, the CT values decreased significantly in low-, or medium-, but not high-firing threshold neurons in bone cancer rats. For example, in LT neurons, the CT value (in pA) decreased to 20.9 ± 1.5 in MRMT-1-rats from 29.8 ± 2.0, 28.9 ± 2.2, and 28.9 ± 2.4 in naïve, PBS-, and HK-treated rats, respectively (*p *< 0.001, as compared with control groups, two- tailed Student's *t*-test). Similar results were observed in MT neurons, whereas in HT neurons, no significant difference in the CT values was observed in MRMT-1-rats as compared with the control rats (Table 1).

**Table 1:** Intrinsic electrogenic properties of small-sized DRG neurons in naïve, PBS-, HK-, and MRMT-1-rats


		**RMP**	**R_in_**	**CT**	**TP**	**TP rise rate**	**AP amplitude**	**AP overshot**	**AP duration**	**AHP amplitude**	**AHP_80% _duration**	**AP frequency**

		**mV**	**MΩ**	**pA**	**mV**	**v/s**	**mV**	**mV**	**ms**	**mV**	**ms**	**Spikes/s**

CT < 50 pA	Naïve	-50.6 ± 0.5 (29)	654.0 ± 20.1 (33)	29.8 ± 2.0 (29)	-27.6 ± 0.8 (38)	0.8 ± 0.1 (30)	101.8 ± 1.9 (34)	55.9 ± 1.4 (34)	44.6 ± 2.9 (30)	19.2 ± 0.8 (40)	17.6 ± 1.1 (35)	20.5 ± 1.8 (30)
	
	PBS	-50.3 ± 0.7 (18)	634.5 ± 28.5 (13)	28.9 ± 2.2 (18)	-27.8 ± 0.8 (29)	0.7 ± 0.1 (24)	103.4 ± 1.8 (24)	55.5 ± 1.6 (24)	43.2 ± 2.6 (23)	18.9 ± 1.3 (25)	17.7 ± 1.4 (21)	20.6 ± 2.3 (14)
	
	MRMT-1	-45.6 ± 0.5 (52) ***	546.3 ± 15.3 (60) ***	20.9 ± 1.5 (44) ***	-30.7 ± 0.6 (55) **	0.9 ± 0.1 (45) *	94.2 ± 1.9 (55) **	45.7 ± 1.9 (47) ***	34.3 ± 1.4 (54) ***	14.7 ± 0.7 (51) ***	14.6 ± 0.6 (49) *	31.4 ± 1.9 (32) ***

	HK	-50.5 ± 0.6 (13)	638.1 ± 24.9 (19)	28.9 ± 2.4 (13)	-26.6 ± 1.0 (23)	0.7 ± 0.1 (13)	102.6 ± 2.6 (16)	54.7 ± 1.7 (16)	44.7 ± 3.1 (16)	19.7 ± 1.3 (11)	18.2 ± 1.7 (13)	20.3 ± 2.6 (12)
	
	Naïve	-51.7 ± 1.1 (12)	593.0 ± 22.3 (14)	85.0 ± 3.3 (13)	-20.1 ± 0.7 (19)	1.9 ± 0.2 (11)	104.7 ± 3.4 (17)	55.1 ± 1.9 (17)	24.5 ± 2.1 (15)	19.7 ± 1.5 (16)	10.6 ± 1.4 (16)	10.5 ± 1.7 (15)
	
CT50 ~100 pA	PBS	-51.1 ± 0.6 (7)	594.6 ± 25.4 (6)	86.4 ± 4.0 (7)	-19.9 ± 1.5 (6)	2.0 ± 0.3 (5)	105.1 ± 4.3 (6)	56.5 ± 3.1 (6)	24.0 ± 1.9 (6)	20.2 ± 2.6 (7)	11.2 ± 1.7 (7)	13.8 ± 2.2 (6)
	
	HK	-52.6 ± 1.5 (11)	596.9 ± 26.4 (7)	85.5 ± 2.7 (11)	-20.0 ± 1.1 (10)	2.0 ± 0.2 (10)	104.0 ± 2.4 (10)	56.0 ± 2.6 (9)	24.0 ± 1.7 (8)	19.7 ± 2.1 (8)	10.7 ± 1.5 (9)	12.2 ± 3.4 (9)
	
	MRMT-1	-48.6 ± 0.5 (16) **	486.5 ± 25.8 (17) **	72.2 ± 4.0 (16) *	-23.4 ± 1.0 (17) **	2.3 ± 0.2 (14)	98.7 ± 2.6 (17)	46.1 ± 2.9 (15) *	22.1 ± 1.2 (17)	14.4 ± 1.3 (17) *	11.4 ± 1.0 (17)	19.3 ± 3.2 (12) *

CT > 100 pA	Naïve	-54.6 ± 2.1 (7)	550.5 ± 40.8 (9)	157.9 ± 12.6 (7)	-16.6 ± 1.0 (8)	5.4 ± 1.1 (5)	103.7 ± 3.6 (7)	54.1 ± 3.3 (5)	17.5 ± 3.2 (6)	10.8 ± 1.3 (8)	6.3 ± 0.6 (6)	7.1 ± 3.4 (8)
	
	PBS	-54.1 ± 1.9 (7)	556.6 ± 35.3 (7)	158.6 ± 14.6 (7)	-18.8 ± 0.8 (9)	5.1 ± 0.7 (5)	107.9 ± 4.8 (7)	56.7 ± 3.7 (6)	15.1 ± 1.1 (6)	10.9 ± 1.8 (6)	6.1 ± 0.5 (5)	6.8 ± 2.9 (8)
	
	HK	-54.2 ± 1.6 (6)	552.0 ± 28.5 (6)	163.3 ± 13.2 (6)	-17.0 ± 1.2 (7)	5.1 ± 1.2 (5)	106.9 ± 6.3 (7)	56.2 ± 2.4 (6)	17.0 ± 4.6 (6)	10.6 ± 1.8 (6)	6.3 ± 0.6 (6)	7.3 ± 2.6 (6)
	
	MRMT-1	-51.2 ± 0.9 (13)	508.1 ± 24.9 (12)	161.7 ± 16.6 (9)	-20.2 ± 1.4 (9)	5.0 ± 0.9 (7)	103.3 ± 4.9 (10)	54.9 ± 2.6 (8)	13.9 ± 1.4 (9)	9.0 ± 1.39 (10)	6.3 ± 0.9 (7)	10.0 ± 4.3 (8)

Values are presented as mean ± S.E.M with the number given in parentheses. *p < 0.05, **p < 0.01,***p < 0.001, as compared with HK groups, two-tailed Student's *t*-test. RMP: resting membrane potential; R_in_: membrane input resistance; CT: the depolarized current threshold for evoking the 1^st ^action potential; TP: threshold potential; AHP: afterhyperpolarization; AHP_80% _duration: AHP duration at 80% repolarization (AHP_80%_);

AP: action potential. Note that implantation of MRMT-1 tumor cells into the tibial canal in rats increased the cells excitability in low (CT < 50 pA)-, medium (CT: 50 - 100 pA)-, but not high (CT > 100 pA)-firing threshold DRG neurons as assessed by the intrinsic electrogenic parameters.

Similarly, the membrane input resistance (R_in_) also decreased markedly in LT-, MT-, but not HT-neurons in bone cancer rats as compared with control rats. In LT neurons, the mean R_in _(in MΩ) was 654.0 ± 20.1, 634.5 ± 28.5, and 638.1 ± 24.9 in naïve, PBS-, and HK-treated rats, respectively; whereas it decreased dramatically to 546.3 ± 15.3 in MRMT-1-rats (*p *< 0.001, as compared with control groups, two-tailed Student's *t*-test). The mean R_in _also decreased significantly in MT neurons (*p *< 0.05), but no significant decrease was observed in HT neurons in MRMT-1-rats (*p *> 0.05) (Table 1).

### Depolarization in resting membrane potential (RMP) and reduction in action potential threshold (TP) in small-sized DRG neurons in MRMT-1-rats

**Figure 3:**
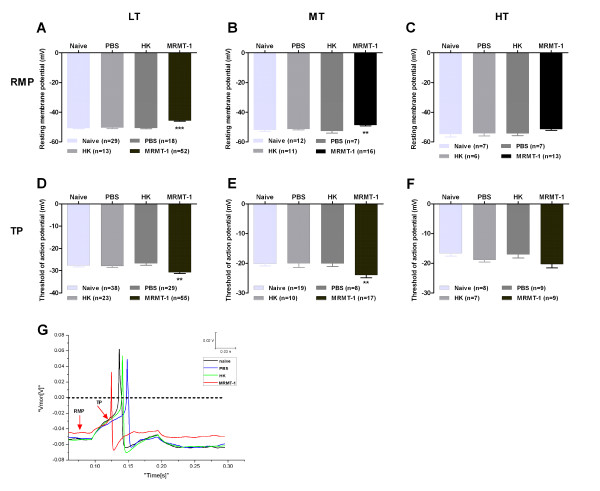
**Changes in resting membrane potentials (RMP) and action potential threshold (TP) of small-sized DRG neurons in rats with intra-tibial injections of MRMT-1 cells**. **(A) **- **(C)**: Summary of the absolute values of RMP in low-, medium-, and high-firing threshold neurons in naïve, PBS-, HK-, and MRMT-1-rats. (**D**) - (**F**): Summary of the absolute values of TP in naïve, PBS-, HK-, and MRMT-1-rats. *p < 0.05, **p < 0.01,***p < 0.001, as compared with HK groups, two-tailed Student's *t*-test. LT, MT, and HT mean low-, medium-, and high-firing threshold neurons, respectively. (**G**): Representative of action potentials evoked by 100-ms, 25-pA depolarizing current pulse, recorded from low-firing threshold DRG neurons. The red trace represents the discharges recorded from a representative small DRG neuron in a MRMT-1-implanted rat. Note that implantation of MRMT-1 tumor cells into the tibial canal in rats caused a depolarizing shift of RMP and a reduction of TP in small-sized DRG neurons.

Implantation of MRMT-1 tumor cells into the tibial canal in rats caused a more depolarized RMP and lowered AP threshold (TP) in LT-, MT-, but not HT-neurons. Therefore, the difference decreased between RMP and TP. As shown in Figure 3, in LT neurons, a more depolarized RMP (in mV) in MRMT-1-rats (-45.6 ± 0.5) was observed than that in naïve (-50.6 ± 0.5), PBS (-50.3 ± 0.7)-, and HK (-50.5 ± 0.6)-treated rats (p < 0.001, as compared with control groups, two-tailed Student's *t*-test). Similar depolarization of RMP was also observed in MT (p < 0.01), but not in HT (p > 0.05) neurons in MRMT-1-rats.

On the contrary, a significantly lowered TP (in mV) was observed in MRMT-1-rats (-30.7 ± 0.6) as compared with those in naïve (-27.6 ± 0.8), PBS (-27.8 ± 0.8)-, and HK (-26.6 ± 1.0)-treated rats in LT or MT neurons (*p *< 0.01), but not in HT neurons (*p *> 0.05), indicating a reduction of AP threshold in these neurons in bone cancer rats. Additionally, the rise rate of TP ((RMP-TP)/duration from RMP to TP) also increased obviously in LT (*p *< 0.05) but not in MT or HT neurons (*p *> 0.05) in MRMT-1-rats (Table 1).

### Decreases in amplitude, overshot, and duration of evoked action potentials in small-sized DRG neurons in MRMT-1-rats

As shown in Table 1, implantation of MRMT-1 tumor cells into the tibial canal in rats significantly decreased the parameters of evoked action potentials (AP) such as amplitude (94.2 ± 1.9 mV vs. 102.6 ± 2.6 mV, *p *< 0.01), overshot (45.7 ± 1.9 mV vs. 54.7 ± 1.7 mV, *p *< 0.001), and duration (34.3 ± 1.4 ms vs. 44.7 ± 3.1 ms, *p *< 0.001) in LT neurons (MRMT-1 group vs. HK group, two-tailed Student's t-test). Except for the decreased overshot of AP in MT neurons (46.1 ± 2.9 mV vs. 56.0 ± 2.6 mV, *p *< 0.05), however, those parameters were not significantly decreased in MT and HT neurons in MRMT-1-rats.

### Decreases in amplitude and duration of afterhyperpolarization (AHP) of evoked action potentials in small-sized DRG neurons in MRMT-1-rats

**Figure 4:**
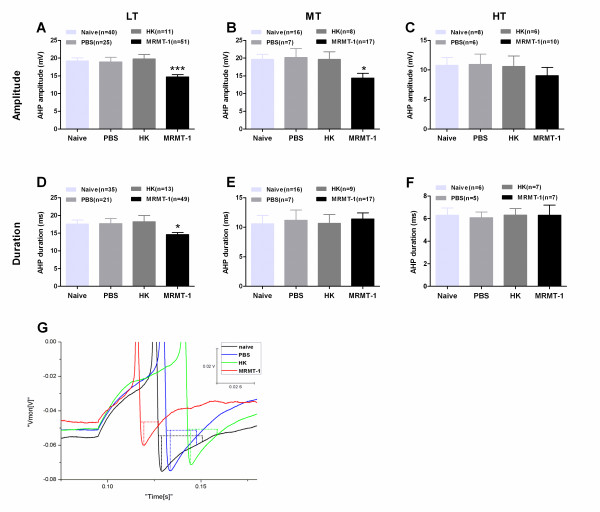
**Changes in the amplitude and duration of afterhyperpolarization (AHP) of action potentials recorded from small-sized DRG neurons in rats with intra-tibial injections of MRMT-1 cells**. (**A**) - (**C**): Summary of the AHP amplitude of action potentials in LT, MT, and HT neurons in naïve, PBS-, HK-, and MRMT-1-rats. (**D**) - (**F**): Summary of the AHP duration at 80% repolarization (AHP_80%_) in naïve, PBS-, HK-, and MRMT-1-rats. *p < 0.05,***p < 0.001, as compared with HK groups, two-tailed Student's *t*-test. LT, MT, and HT mean low-, medium-, and high-firing threshold neurons, respectively. (**G**): Representative of the AHP amplitude and duration of action potentials evoked by 100-ms, 40-pA depolarizing current pulse, recorded from low-firing threshold DRG neurons. The red trace represents the discharges recorded from a representative small DRG neuron in a MRMT-1-implanted rat. The vertical dot line to trough shows the AHP amplitude, and the horizontal dot line shows the AHP_80%_. Action potentials have been amputated due to high gain. Note that implantation of MRMT-1 tumor cells into the tibial canal in rats decreased the amplitude and duration of afterhyperpolarization (AHP) in small-sized DRG neurons.

Apart from the decreased amplitude, overshot, and duration of evoked action potentials, other parameters such as amplitude and duration of the AHP also decreased in LT neurons in MRMT-1-rats. As shown in Table 1 and Figure 4, the AHP amplitude (in mV) decreased dramatically in LT neurons in MRMT-1-rats (14.7 ± 0.7) by contrast with that in naïve (19.2 ± 0.8), PBS (18.9 ± 1.3)-, and HK (19.7 ± 1.3)-treated rats (*p *< 0.001, two-tailed Student's *t*-test). The AHP amplitude also decreased obviously in MT (*p *< 0.05)-, but not in HT (*p *> 0.05)-neurons in bone cancer rats as compared to control rats. Similarly, the AHP duration (in ms) at 80% repolarization (AHP80%) also shortened obviously in LT neurons in MRMT-1-rats (14.6 ± 0.6) in contrast to naïve (17.6 ± 1.1), PBS (17.7 ± 1.4)-, and HK (18.2 ± 1.7)-treated rats (*p *< 0.05, two-tailed Student's *t*-test). No significant decrease was observed in MT and HT neurons in bone cancer rats.

### Increase in firing frequency of evoked action potentials in small-sized DRG neurons in MRMT-1-rats

**Figure 5:**
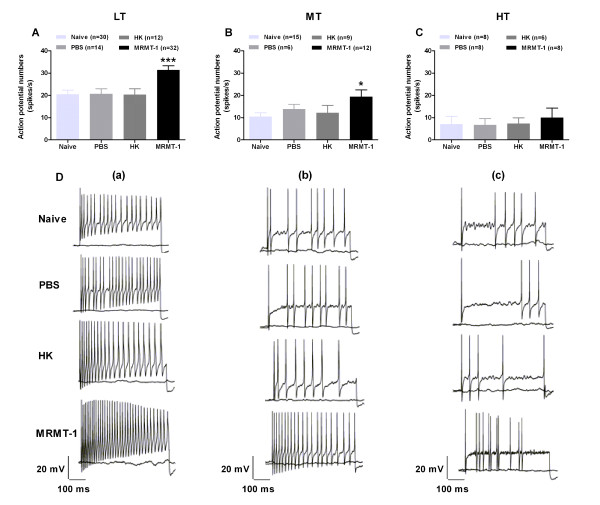
**Changes in the firing frequency of evoked action potentials recorded from small-sized DRG neurons in rats with intra-tibial injections of MRMT-1 cells**. (**A**) - (**C**): Summary of the action potential numbers (spikes/s) evoked by a 1-s, 300-pA depolarizing current pulse in LT, MT, and HT neurons in naïve, PBS-, HK-, and MRMT-1-rats. *P < 0.05, ***P < 0.001, as compared with HK groups, two-tailed Student's *t*-test. (**D**): Representative of evoked action potentials recorded from LT (**a**), MT (**b**), and HT (**c**) neurons. LT, MT, and HT mean low-, medium-, and high-firing threshold neurons, respectively. Note that implantation of MRMT-1 tumor cells into the tibial canal in rats increased the firing frequency of evoked action potentials in LT and MT, but not HT neurons.

Implantation of MRMT-1 tumor cells into the tibial canal in rats increased the firing frequency of small-sized DRG neurons evoked by a constant (1 s, 300 pA) depolarizing current pulse. As shown in Table 1 and Figure 5, the firing frequency of evoked action potentials (in spikes/s) increased markedly in LT neurons in MRMT-1-rats (31.4 ± 1.9) in contrast to naïve (20.5 ± 1.8), PBS (20.6 ± 2.3)-, and HK (20.3 ± 2.6)-treated rats (*p *< 0.001, two-tailed Student's *t*-test). The firing frequency also increased obviously in MT (*p *< 0.05)-, but not in HT (*p *> 0.05)-neurons in bone cancer rats as compared to control rats.

### No significant changes in excitability and intrinsic membrane properties of small-sized DRG neurons in MRMT-1-rats without allodynia

To clarify whether the alterations in intrinsic membrane properties and the enhanced excitability of small-sized DRG neurons were associated with behavioral changes in bone cancer rats, we also examined the aforementioned properties in MRMT-1-rats without allodynia. As our expectation, no significant changes in excitability and intrinsic membrane properties were observed in small-sized DRG neurons in MRMT-1-rats without allodynia (Additional file 1: Figure S1 and Additional file 3: Table S1).

### Characterization of the isolated small DRG neurons in rats

**Figure 6:**
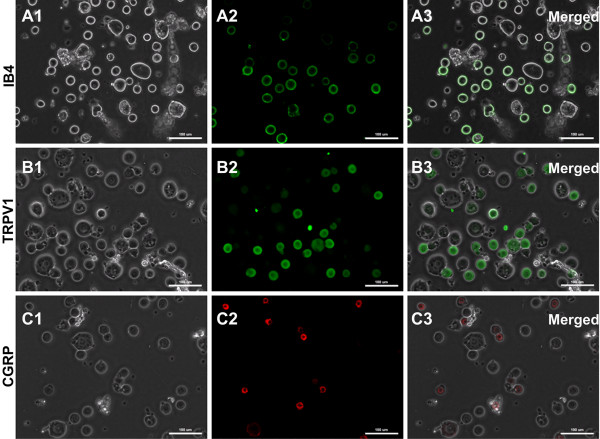
**Immunofluorescent staining of IB4, CGRP and TRPV1 in isolated DRG neurons**. (**A1**) - (**A3**): IB4-FITC staining to living cells. (**B1**) - (**C3**): immunofluorescent staining of TRPV1 (**B1 **- **B3**) and CGRP (**C1 **- **C3**) in isolated DRG neurons. (**A1**) - (**C1**): viewed under bright field microscope. (**A2**) - (**C2**): viewed under fluorescence microscope. (**A3**) - (**C3**): merged. Scale bar: 100 μm.

**Figure 7:**
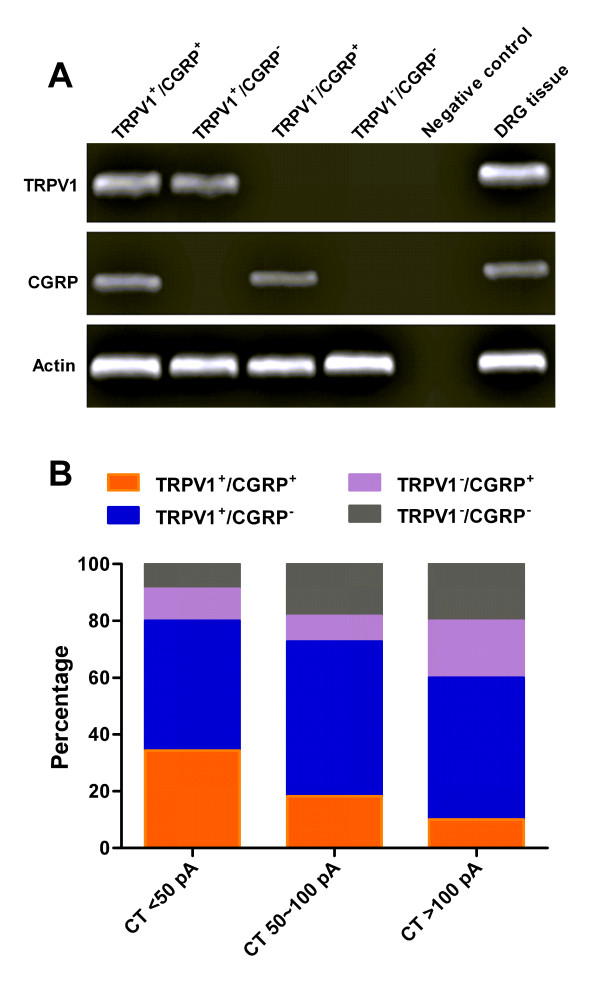
**Single-cell reverse- transcriptase PCR analysis for TRPV1 and CGRP mRNA expression in each recorded LT, MT, and HT DRG neuron**. (**A**): Reverse transcription-PCR analysis of RNA isolated from single DRG neuron using primer pairs for TRPV1, CGRP, and actin. Negative control was processed without the addition of primer pairs, while the DRG tissue was used as positive control. (**B**): Summary of TRPV1 and CGRP mRNA expression in total recorded LT (CT < 50 pA), MT (CT 50 ~ 100 pA), and HT (CT > 50 pA) DRG neurons.

To further characterize the small-sized DRG neurons that were examined in our present study, we first did immunofluorescent staining of IB4, CGRP and TRPV1 in isolated DRG neurons, respectively. The results showed that in isolated small DRG neurons obtained from 5 rats for each staining, the IB4-positive neurons were 71.9% (340/473), the TRPV1-positive neurons were 66.1% (257/389), and the CGRP-positive neurons were 27.7% (135/487), indicating that most small-sized DRG neurons recorded in our study were nociceptive neurons (Figure 6). We then did single-cell reverse-transcriptase PCR to detect TRPV1 and CGRP mRNA expression in each recorded small DRG neuron after current-clamp recording. As shown in Figure 7 and Additional file 4: Table S2 (supplementary data), in 56 recorded small DRG neurons, 75.0% (42/56) were TRPV1^+ ^neurons, 39.3% (22/56) were CGRP^+ ^neurons, whereas only 12.5% (7/56) were TRPV1^-^/CGRP^- ^neurons; among those, 35 were low (LT)-firing threshold (CT < 50 pA) neurons (62.5%), 11 were medium (MT)-firing threshold (CT 50 ~ 100 pA) neurons (19.6%), and 10 were high (HT)-firing threshold (CT > 50 pA) neurons (17.9%). In the 35 LT neurons, TRPV1^+^/CGRP^+ ^neurons were 34.3%, TRPV1^+^/CGRP^- ^neurons were 45.7%, TRPV1^-^/CGRP^+ ^neurons were 11.4%, TRPV1^-^/CGRP^-^neurons were 8.6%; In the 11 MT neurons, TRPV1^+^/CGRP^+ ^neurons were 18.2%, TRPV1^+^/CGRP^- ^neurons were 54.5%, TRPV1^-^/CGRP^+ ^neurons were 9.1%, while TRPV1^-^/CGRP^- ^neurons were 18.2%; Whereas, in the 10 HT neurons, TRPV1^+^/CGRP^+ ^neurons were 10%, TRPV1^+^/CGRP^- ^neurons were 50%, TRPV1^-^/CGRP^+ ^neurons were 20%, while TRPV1^-^/CGRP^- ^neurons were increased to 20%, indicating that TRPV1, CGRP or both were expressed in most LT and MT neurons, whereas the cells that expressed TRPV1 or CGRP decreased significantly in HT neurons. Actually, these results also revealed that the current threshold (CT) of action potential was likely related to TRPV1 mRNA expression in each small DRG neuron, e.g., the less TRPV1 mRNA expression, the higher CT in a DRG neuron.

### Intrathecal administration of tetrodotoxin (TTX) inhibited the tumor-evoked mechanical allodynia and thermal hyperalgesia in bone cancer rats

**Figure 8:**
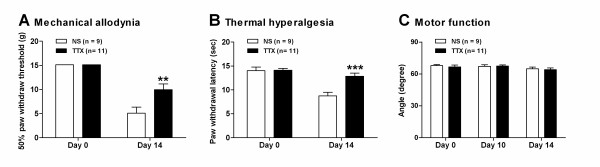
**Effects of intrathecal (i.t.) injection of tetrodotoxin (TTX) on pain behaviors and motor function in rats with intra-tibial injections of MRMT-1 cells**. (**A**): 50% paw withdrawal threshold (PWT); (**B**): paw withdrawal latency (PWL); (**C**): inclined-plate test. Note that i.t. injection of TTX inhibited the tumor-evoked (as assessed by 50% PWT) and thermal hyperalgesia (as assessed by PWL), but did not affect the motor function in bone cancer rats. **p < 0.01, ***p < 0.001, as compared with i.t. injection of saline rats, two-way ANOVA followed by Bonferroni post-hoc test.

Finally, we investigated whether intrathecal (i.t.) administration of tetrodotoxin (TTX), which was considered to be able to block the neurons excitation, could inhibit the tumor-evoked mechanical allodynia and thermal hyperalgesia in bone cancer rats. Indeed, our results demonstrated that i.t. injection of TTX (10 μg/kg, once a day) significantly inhibited the tumor-evoked mechanical allodynia (as assessed by 50% PWT) and thermal hyperalgesia (as assessed by PWL) in MRMT-1-rats. As shown in Figure 8A and 8B, both of the tumor-induced decreases in 50% paw withdrawal threshold (PWT) to von Frey filaments (9.96 ± 1.2 g versus 5.09 ± 1.3 g, TTX versus NS, *P *< 0.01) and in paw withdrawal latency (PWL) to radiant heat (12.82 ± 0.7 s versus 8.75 ± 0.8 s, TTX versus NS, *P *< 0.001) were dramatically inhibited in TTX-treated rats as compared with those in saline-treated rats. To further clarify whether i.t. injection of TTX at our experimental dose affected the animal's motor function, we performed inclined-plate test to measure rats' motor function before and after TTX injection, respectively. The results showed that i.t. injection of TTX had no obvious motor dysfunction in contrast to vehicle injection (*p *> 0.05, two-way ANOVA followed by Bonferroni post-hoc test, Figure **8**C). These results suggested that suppression of DRG neurons excitability likely inhibited the tumor-evoked mechanical allodynia and thermal hyperalgesia in bone cancer rats.

## Discussion

### Tumor-evoked hyperalgesia and sensitization of small-sized DRG neurons

Consistent with previous reports [28,29], we presently observed that implantation of MRMT-1 tumor cells into the tibial canal in rats produced significant mechanical and thermal hyperalgesia in the ipsilateral hind paw, indicating the development of tumor-induced bone cancer pain. In addition, we also found that implantation of tumor cells provoked spontaneous discharges and tonic excitatory discharges evoked by 1-s, 300-pA depolarizing current pulse in small-sized DRG neurons, suggesting a sensitization or hyperexcitability of primary sensory neurons appear to occurrence in cancer condition. Our present results are consistent with previous reports that the C-fiber nociceptors in mice with tumor-evoked hyperalgesia exhibited ongoing activity and sensitization to heat stimuli [8]. These findings provided strong evidence to the notion that sensitization of primary afferent DRG neurons innervating tissue near the tumor likely contribute to the persistent pain and hyperalgesia under cancer condition [8-10].

It has been suggested that neuronal sensitization refers to an increase in excitability characterized by spontaneous activity, lowered response thresholds, and increased response to suprathreshold stimuli [8], all of which were observed in the present study. The most striking difference in the responses of small-sized DRG neurons in tumor-bearing rats was the high incidence of spontaneous discharges and tonic excitatory discharges evoked by a depolarizing current pulse. The depolarizing current-induced tonic responses of small-sized DRG neurons in bone cancer rats (Figure 2B) suggest the appearance of neuronal hyperexcitability, while the spontaneous activity is probably involved in the development of spontaneous (ongoing) pain behaviors exhibited in murine models of bone cancer pain [7,9]. Apart from the tumor-evoked sensitization of C nociceptors [8], sensitization of wide dynamic range (WDR), but not high threshold (HT), nociceptive dorsal horn neurons to mechanical, heat, and cold stimuli has also been observed in mice with tumor-evoked hyperalgesia [6]. Ongoing activity in unmyelinated and myelinated nociceptors has also been associated with nerve injury [30,31] or tissue inflammation [32-34]. Clinical and experimental observations implicate spontaneous activity as a probable substrate of persistent neuropathic pain and progressive degenerative neuropathies [35,36]. Taken together, our present study strongly suggests that sensitization of primary sensory neurons contributes to the tumor-induced hyperalgesia and ongoing pain.

### Tumor-evoked hyperexcitability of small-sized DRG neurons associated with alterations in the intrinsic membrane properties

It has been demonstrated that following nerve injury there is an enhancement of excitability in C cells of DRG as evidenced by a reduction in AP threshold [13]. Specifically, the nerve injury-induced increase in excitability, including depolarization of RMP, decreased AHP and rheobase, and repetitive firing during depolarization, is pronounced after direct injury by axotomy of L5 neurons but is absent in adjacent L4 neurons after spinal nerve ligation [37]. In guineapig DRG neurones, decreased somatic AP and AHP durations are seen in nociceptive but not low threshold mechanoreceptive (LTM) neurones 2 or 4 days after complete Freund's adjuvant (CFA) treatment, suggesting that the alterations in membrane properties of nociceptors may permit higher discharge frequencies, thus contributing to inflammatory hyperalgesia [21]. In line with these observations, we presently found significant alterations of intrinsic membrane properties in small-sized DRG neurons in bone cancer rats, of which including: 1) depolarized resting membrane potential (RMP); 2) decreased input resistance (R_in_); 3) a marked reduction in AP current threshold (CT) and AP voltage threshold (TP); 4) a dramatic decrease in amplitude, overshot, and duration of evoked action potentials as well as in amplitude and duration of AHP; and 5) a significant increase in the firing frequency of evoked action potentials. In this study, we only examined the small-sized DRG neurons, most of which are considered as nociceptive primary sensory neurons. Indeed, the immunostaining results showed that in acute isolated small DRG neurons, most neurons were IB4-positive (71.9%), or expressed TRPV1 (66.1%) or CGRP (27.7%). Additionally, the single-cell reverse-transcriptase PCR also demonstrated that in all recorded small DRG neurons, 75.0% were TRPV1^+ ^neurons, 39.3% were CGRP^+ ^neurons, whereas only 12.5% were TRPV1^-^/CGRP^- ^neurons. These results suggest that most small-sized DRG neurons recorded in our experiments are nociceptive neurons.

Somata of the small-sized DRG neurons were classified by their diameters (≤ 26 μm) and C_m _(≤ 45 pF) as described in previous reports [38,39]. In the present study, we observed three types of small DRG neurons i.e. low (LT)-, medium (MT)-, and high-firing threshold (HT)-DRG neurons as classified by the AP current threshold (CT), and all of the alterations in intrinsic membrane properties appeared to occurrence in small-sized DRG neurons of LT, or MT, but not HT cells in bone cancer rats. In addition, by the single-cell reverse-transcriptase PCR analysis, we also found that the current threshold (CT) of action potential is likely related to TRPV1 mRNA expression in each small DRG neuron, e.g., the less TRPV1 mRNA expression, the higher CT in a DRG neuron (Figure 7 and Additional file 4: Table S2). Therefore, the classification of small-sized DRG neurons by the current threshold of action potentials is probably able to reflect their functional characters. For instance, the lower CT of a small DRG neuron suggests a higher TRPV1 mRNA expression, and more likely represents as a nociceptive neuron. Although the mechanisms underlying the development of bone cancer pain are largely unclear, the decreased CT and increased firing frequency of evoked action potentials implicated that the hyperexcitability of small-sized DRG neurons likely contributed to the tumor-induced hyperalgesia [2,40]. We speculate that the depolarized RMP and the reduced TP may account for the decreased CT in small -sized DRG neurons as observed in tumor-bearing rats. Our observation of a reduction in AP threshold in small-sized DRG neurons is consistent with previous reports that nerve injury results in an enhancement of excitability in C cells associated with a reduction in AP threshold, and that one possible explanation for this reduction is a preferential increase in expression of TTX-sensitive sodium currents [13,41]. For most of the membrane properties recorded from small DRG neurons with our preparation, the decreased input resistance (R_in_) in bone cancer rats is consistent with that of a decreased R_in _in C-type neurons in mouse DRG after partial colonic obstruction [42], but inconsistent with that of increase in A cells, or no alteration in C cells of axotomized rat DRGs [13,18]. The wide range of changes in R_in _suggests the possibility of different underlying mechanisms. For example, R_in _could be decreased by a depolarization of the RMP and an increase in I_h _currents [43] or increased by the inhibition of K^+ ^currents [44], or unaffected by an enhancement of Na^+ ^currents [13,45,46] or reduction of Ca^2+ ^currents [47]. Therefore, the depolarization of the RMP is likely to underlie the decreased R_in _in small-sized DRG neurons in MRMT-1 rats.

Other changes in membrane properties of small DRG neurons in bone cancer rats also include a dramatic decrease in amplitude, overshot, and duration of evoked action potentials as well as in amplitude and duration of AHP. These results are supported by the findings that shorter duration of APs in C and Aδ-fibre nociceptive neurons with decreased rise times in C-fibre nociceptors, decreased fall times in C and Aδ-fibre units, and shorter duration of AHP_80% _in Aδ- and Aα/β-fibre units take place in nociceptive DRG neurons during CFA-induced peripheral inflammation [21]. It has been accepted that the changes in membrane properties include alterations in level or activity of proteins involved in pathways regulating cation channels, e.g. channel subunit, cAMP-dependent protein kinases, protein kinase C or G proteins [48,49]. Together with previous findings that the AP rise time is faster in C-fibre nociceptors after CFA-induced inflammation [21] and the voltage-gated sodium channels are kinetically the fastest channel type contributing to the AP, we infer that an overall increase in sodium channel density, or relative alterations in levels of sodium channels with differing kinetics [50] or modulation of pre- existing sodium channels [26] likely contribute to the reduced AP duration under cancer condition. Alternatively, the decreased AP amplitude and overshot under cancer condition are proposed due to the depolarization in RMP, which reduced the driving force of voltage-gated sodium currents [26,27].

Several studies have revealed that the rate of decay (i.e. duration) of the AHP is depended on Ca^2+ ^influx that affects the activation Ca^2+^-dependent K^+ ^channels (K Ca) [51-53]. Reduction of AP broading (i.e. duration) during a train of APs would result in a reduced Ca^2+ ^influx and less activation of the Ca^2+^-dependent K^+ ^channels, and therefore lead to a reduced AHP. Interestingly, Kvβ1.1-deficient mice, which show reduced AP broading, also have a reduced AHP [54], whereas diabetic rats, which show increased AP broading, have an increased AHP [55]. Thus the reduced AP duration in small-sized DRG neurons provides an alternative explanation for the reduced AHP under cancer condition. Since AHP limits firing frequency, a reduction in the AHP would be expected to increase the number of APs in response to stimuli [53,56]. We therefore suggest that the attenuation in AHP may reduce the inhibition on firing, and thus lead to the enhancement in intrinsic excitability under cancer condition [57]. Taken together, the decrease in amplitude, overshot, and duration of evoked action potentials as well as in amplitude and duration of AHP likely contribute to the increased firing frequency of evoked action potentials in small DRG neurons in MRMT-1 rats.

### Blockade of DRG neurons activity by TTX inhibited the tumor-evoked mechanical allodynia and thermal hyperalgesia in bone cancer rats

To further clarify whether enhanced excitability of primary sensory neurons contributes to the development of peripheral sensitization and bone cancer pain, we examined the effects of tetrodotoxin (TTX) on the tumor-evoked mechanical allodynia and thermal hyperalgesia in bone cancer rats. We found that i.t. injection of TTX significantly inhibited the tumor-evoked mechanical allodynia (as assessed by 50% PWT) and thermal hyperalgesia (as assessed by PWL) in MRMT-1-rats. Previously, using extracellular microelectrode recordings from DRG that reflect the activity of single DRG neuron, Omana-Zapata et al. [58] have reported that systemic administration of TTX produces a dose-dependent reduction in DRG neurons activity. A significant decrease in DRG neurons activity is obtained at the dose of 1 μg/kg, with complete inhibition usually achieved at 10 μg/kg, and the ED_50 _value for TTX in the DRG is 4.3 μg/kg. Based on these observations, we therefore applied TTX at the dose of 10 μg/kg, which was considered to be able to completely block the DRG neurons activity, but can not significantly inhibit the dorsal horn neurons activity, because the inhibitory effect of TTX on DRG neurons activity is markedly greater than that on dorsal horn neurons activity, e.g. the ED_50 _value for TTX in the dorsal horn is 36.2 μg/kg [58]. Indeed, we also found that i.t. injection of TTX at 10 μg/kg almost completely inhibited the DRG neurons activity with little effect on the dorsal horn neurons activity (data not shown). Our present results are consistent with previous reports that a blockade of DRG neurons activity with local application of lidocaine to the DRG alleviated the mechanical hyperalgesia and tactile allodynia in rat modesl of neuropathic pain [59-61]. Finally, to further elucidate whether the inhibitory effects of TTX on tumor-evoked mechanical allodynia and thermal hyperalgesia resulted from the TTX-induced motor dysfunction, we performed inclined-plate test to measure rats' motor function before and after TTX injection, respectively. Our results showed that i.t. injection of TTX at our experimental dosage had no obvious motor dysfunction in contrast to vehicle injection, indicating that the inhibitory effects of TTX on tumor-evoked mechanical allodynia and thermal hyperalgesia likely resulted from the TTX-induced inhibition on DRG neurons activity. Taken together, these results provide in vivo evidence to the notion that the enhanced excitability of primary sensory neurons under cancer condition likely contributes to the development of peripheral sensitization and bone cancer pain, although we can not conclude that cancer is unique in the mechanisms that enhance the DRG neurons excitability as compared to other injuries.

## Conclusions

In summary, our present study showed that: (1) implantation of MRMT-1 tumor cells into the tibial canal in rats produced significant mechanical and thermal hyperalgesia in the ipsilateral hind paw; (2) implantation of tumor cells induced an enhanced excitability of small-sized DRG neurons that is probably as results of alterations in intrinsic membrane properties of these neurons; (3) Immunofluorescent staining and single-cell reverse-transcriptase PCR revealed that in isolated small DRG neurons, most neurons were IB4-positive, or expressed TRPV1 or CGRP, indicating that most recorded small DRG neurons were nociceptive neurons; (4) blockade of DRG neurons activity by TTX inhibited the tumor-evoked mechanical allodynia and thermal hyperalgesia in bone cancer rats, implicating that the enhanced excitability of primary sensory neurons underlied the development of bone cancer pain. Thus, this study suggests that alterations in intrinsic membrane properties associated with the hyperexcitability of primary sensory neurons likely contribute to the peripheral sensitization and tumor-induced hyperalgesia under cancer condition.

## Methods

### Animals

Female Sprague-Dawley rats weighing 150 - 180 g at the beginning of the experiment were used (Department of Experimental Animal Sciences, Peking University Health Science Center). The rats were housed in separated cages with free access to food and water. The room temperature was kept at 24 ± 1°C under natural light-dark cycle. All animal procedures were carried out in accordance with the guidelines of the International Association for the Study of pain [62] and were approved by the Animal Care and Use Committee of Peking University.

### Bone cancer pain model

#### Implantation of tumor cells

A rat model of bone cancer pain was established by intra-tibial injections of syngeneic MRMT-1 rat mammary gland carcinoma cells as previously described [28]. Briefly, after anesthetized with chloral hydrate (0.3 g/kg, i.p.), the rat left tibia was carefully exposed, and a 23-gauge needle was inserted into the intramedullary canal of the bone. It was then removed and replaced with a long thin blunt needle attached to a 10-μl Hamilton syringe containing the medium to be injected. A volume of 4 μl MRMT-1 rat mammary gland carcinoma cells (4 × 10^4^), inactivated (heat-killed) carcinoma cells or vehicle (phosphorylated buffer solution, PBS) was injected into the tibial bone cavity. Following injection the site was sealed with bone wax, and the wound was finally closed. None of the animals showed signs of motor dysfunction after implantation of tumor cells.

#### Assessment of mechanical allodynia

Mechanical allodynia, as a behavioral sign of bone cancer pain, was assessed by measuring 50% paw withdrawal threshold (PWT) as described in our previous reports [63,64]. The 50% PWT in response to a series of von Frey filaments (Stoelting, Wood Dale, IL, USA) was determined by the Up and Down method [65]. The rat was placed on a metal mesh floor covered with an inverted clear plastic cage (18 × 8 × 8 cm) and allowed a 20-min period for habituation. Eight von Frey filaments with approximately equal logarithmic incremental (0.224) bending forces were chosen (0.41, 0.70, 1.20, 2.00, 3.63, 5.50, 8.50, and 15.10 g). Each trial started with a von Frey force of 2.00 g delivered perpendicularly to the plantar surface of the left hindpaw for about 2 - 3 s. An abrupt withdrawal of the foot during stimulation or immediately after the removal of the hair was recorded as a positive response. Whenever there was a positive or negative response, the next weaker or stronger filament was applied, respectively. This procedure was done until six stimuli after the first change in response had been observed. The 50% PWT was calculated using the following formula: $50\%\;\text{PWT}=10\begin{array}{c} \left(\text{X}+\text{k}\delta \right)\\ \text{f} \end{array}/10^{4}$, where X_f _is the value of the final von Frey filament used (in log units), k is a value measured from the pattern of positive/negative responses, and δ = 0.224, which is the average interval (in log units) between the von Frey filaments [66]. If an animal responded to the lowest von Frey filament, a value of 0.25 g was assigned. If an animal did not respond to the highest von Frey filament, the value was recorded as 15.0 g. Testing sessions were performed on the day before injection and day 3, 7, 10, 14, 21 and 28 after tumor cells or sham injection. The mechanical allodynia was assessed by measuring 50% PWT of ipsilateral hind paw in tumor cells inoculated and sham-operated rats, respectively. In rats, mechanical allodynia is assessed by measuring 50% paw withdrawal threshold (PWT) to von Frey filaments, and an allodynic rat is defined as that the 50% PWT is less than 4.0 g, i.e. withdrawal in response to non-noxious tactile stimulus [17].

#### Assessment of thermal hyperalgesia

Thermal hyperalgesia of the hind paws was tested as described by our previous report [63]. Rats were allowed to acclimate for a minimum of 30 min within acrylic enclosures on a clear glass plate maintained at 30°C. A radiant heat source was focused onto the plantar surface of the hind paw. Measurements of paw withdrawal latency (PWL) were taken by a timer that was started by the activation of the heat source and stopped when withdrawal of the paw was detected with a photodetector. A maximal cut-off time of 30 s was used to prevent unnecessary tissue damage. Three measurements of PWL were taken for each hind paw and were averaged as the result of each test session. The hind paw was tested alternately with greater than 5 min intervals between consecutive tests.

Approximately 90% of rats implanted with tumor cells exhibited sufficient mechanical allodynia and thermal hyperalgesia, and were used in the electrophysiological studies. Electrophysiological studies of DRG neurons in tumor-bearing rats were performed 10 - 28 days after implantation as mechanical allodynia and thermal hyperalgesia are maximal during this period (Figure 1).

### Measurement of TTX effects on pain behaviors in bone cancer rats

#### Implantation of intrathecal catheter

Under chloral hydrate (0.3 g/kg, i.p.) anesthesia, implantation of intrathecal cannula was performed as described in our previous report [63]. Briefly, a PE-10 polyethylene catheter was implanted between the L5 and L6 vertebrae to reach the lumber enlargement of the spinal cord. The outer part of the catheter was plugged and fixed onto the skin on closure of the wound. All surgical procedures were performed under sterile conditions. Rats showing neurological deficits after the catheter implantation were euthanized. Drugs or vehicle were intrathecally injected via the implanted catheter in a 20-μl volume of solution followed by 10 μl of normal saline (NS) for flushing. Each injection lasted at least 5 min. After an injection, the needle remained in situ for 2 min before being withdrawn.

#### Measurement of TTX effects

The 50% PWT to von Frey filaments and the PWL to radiant heat were first measured as baseline control before inoculation of tumor cells to rats. Implantation of intrathecal catheter was performed on day 7 after tumor cells inoculation. After assessing the rats' motor function by inclined-plate testing, intrathecal (i.t.) injection of tetrodotoxin (TTX) was performed once a day on day 10 post-inoculation. The administration of TTX lasted for 3 days to day 12 post-inoculation. The 50% PWT to von Frey filaments, the PWL to radiant heat and the inclined-plate test were then measured on day 12, 13, and 14 after tumor cells inoculation. TTX at the dose of 10 μg/kg (in a 20-μl volume of solution), or normal saline in an equal volume, was intrathecally administrated to the rat once a day.

#### Assessment of motor function

Inclined-plate test was used for the assessment of motor function. The rat was placed crosswise to the long axis of an inclined plate. The initial angle of the inclined plate was 50 degrees. The angle was then adjusted in 5-degree increments. The maximum angle of the plate on which the rat maintained its body position for 5 s without falling was determined according to the method reported by Rivlin and Tator [67].

### Whole-cell patch-clamp recording

Neurons were isolated from ipsilateral L4 and L5 DRG of adult rats using methods as previously described [68]. Briefly, freshly dissected ganglia were minced and washed in cold, oxygenated DMEM (Sigma, St. Louis, MO), and were then subjected to collagenase (3 mg/ml, type IA, Sigma) treatment for 45 min, followed by trypsin (2 mg/ml, Type II-S, Sigma) for 15 min at 37°C. The enzymatic reaction was stopped by washing the cells with DMEM containing 10% fetal bovine serum (FBS), and the remaining pieces of ganglia were gently triturated by using a fire-polished glass Pasteur pipette and passed through a 40 μm cell strainer. The suspension was then centrifuged at 800 rpm for 3 min, and the cell pellet was resuspended in fresh DMEM supplemented with 10% FBS. The dissociated cells were placed on poly-D-lysine (0.1 mg/ml, sigma)-treated glass coverslips contained within 24-well sterile tissue culture plates and kept in 5% CO_2 _incubator at 37°C for 2-3 h before recording.

Whole-cell current-clamp recordings were performed at room temperature using an EPC-10 amplifier and Patchmaster software (HEKA, Germany). Patch pipettes were pulled from borosilicate glass capillaries with a tip resistance of 2 - 5 MΩ when filled with internal solution containing (in mM) 80 K-acetate, 30 KCl, 40 HEPES, 3 MgCl_2_, 3 EGTA, and 1 CaCl_2_, adjusted to pH 7.4 with KOH. The external solution contained (in mM) 144 NaCl, 2.5 KCl, 2 CaCl_2_, 0.5 MgCl_2_, 5 HEPES, and 10 Glucose, adjusted to pH 7.4 with NaOH. Action potentials (AP) were measured with pipette and membrane capacitance cancellation, filtered at 2 kHz and digitized at 10 kHz. Series resistance was compensated 70 - 90%. The membrane capacitance (C_m_) was read from the amplifier by software Patchmaster for determining the size of cells.

For current-clamp recording, the cells were held at 0 pA, and the firing threshold of DRG neurons was first measured by a series of 100-ms depolarizing current injection in 5 pA steps from 0 pA to elicit the 1^st ^action potential. To further examine the neurons firing properties, a large depolarizing current injection in 1 s, 300 pA was delivered to elicit the cell generating sufficient firing. In our present study, a depolaring current pulse (1-s, 300 pA) was injected in all neurons, because we tried to ensure that the evoked discharges were elicited under a equal depolaring current pulse in all neurons, so the firing frequency and other parameters of intrinsic membrane properties were comparable between control and experimental DRG neurons. We have demontrated that this depolaring current pulse (1-s, 300 pA) could evoke action potentials firing in all recorded small DRG neurons in our preliminary experiments. A hyperpolarizing current injection in 200 ms, -200 pA was used to measure membrane input resistance (R_in_), which was assessed from the value of the evoked membrane potential divided by the injected hyperpolarizing current (-200 pA). The following values were measured in this study (Additional file 2: Figure S2): resting membrane potential (RMP), the depolarized current threshold for eliciting the 1^st ^action potential (CT), threshold potential (TP), rise rate of TP ((RMP-TP)/duration from RMP to TP), amplitude of action potential (AP), overshot of AP; duration of AP, amplitude of afterhyperpolarization (AHP), AHP duration at 80% repolarization (AHP_80%_), and frequency of AP.

### Immunofluorescent staining of IB4, CGRP and TRPV1 in isolated DRG neurons

#### IB4 staining of isolated DRG neurons

We used IB4-FITC (isolectin B4 conjugated to fluorescein isothiocyanate), which binds to living cells, to distinguish small-sized sensory neurons (soma diameter ≤ 26 μm) capable of binding this lectin from other small neurons. Briefly, the isolated DRG neurons were incubated with IB4-FITC (10 μg/ml, Sigma-Aldrich) for 10 min and rinsed for 2 min, and the IB4-FITC staining was then visualized. A neuron was considered IB4 positive if it had a continuous green ring around the perimeter [39].

#### Immunofluorescent staining of CGRP and TRPV1

For immunofluorescent staining of CGRP and TRPV1 in isolated DRG neurons, the cells were rinsed in 0.1 M phosphate-buffered saline (PBS, pH 7.4) two times, fixed in 4% paraformaldehyde (in 0.1 M PB, pH 7.4) for 15 min, and soaked in 0.25% Triton X-100 (in 0.1 M PB, pH 7.4) for 10 min. After blocking in 5% bovine serum albumin (BSA) for 1 h, the cells were incubated with the following primary antibodies as mouse anti-rat calcitonin gene-related peptide (CGRP, 1:1,000, Sigma) or rabbit anti-rat TRPV1 (1:1000, Santa Cruz Biotechnology) in 1% BSA and 0.3% Triton-X100 (in 0.01 M PBS), over night at 4°C. Control cells were processed without the addition of primary antibody. After three rinses with PBS, cells were incubated with secondary antibodies for 1 h at room temperature and washed three times with PBS, in which the Alexa Fluor 488 goat anti-mouse IgG (H + L) was used for CGRP staining and the Alexa fluor 568 goat anti-rabbit IgG (H + L) was used for TRPV1 staining. Both of them were purchased from Invitrogen and were used at the dilution of 1:1000. Stained cells were viewed and photographed with a CCD camera under a fluorescent microscope (DMIRB, Leica, Germany).

### Single-cell reverse-transcriptase PCR

Single-cell reverse-transcriptase PCR was performed according to the method as previously described [69]. In brief, after current-clamp recording and measurement of the neuron's current threshold (CT), cytoplasm and nucleus of the single recorded cell were harvested into the patch pipette under visual control. The contents of the patch pipette were expelled into a reaction tube containing 2 μl prepared Random primer dilution (0.1 μg/μl, Random primer 1 μl, DEPC·H_2_O 1 μl) and were incubated at 70°C for 5 min. Reverse transcription of mRNA transcripts were initiated by the addition of 18 μl prepared reverse transcription mixture containing: Moloney murine leukemia virus (M-MLV) reverse transcriptase RNase H (-) point mutant (Invitrogen) 0.5 μl (100 U), 5 × M-MLV buffer 4 μl, 10 mM dNTP 0.2 μl, and RNase inhibitor (Rasin) 0.4 μl, adjusted to the final volume (18 μl) with DEPC·H2O, followed by incubation at 37°C for 45 min and 70°C for 10 min. A multiplex PCR protocol was then used to amplify cDNA for TRPV1, CGRP and actin simultaneously. Primers were designed as follows: rat TRPV1, forward GACATGCCACCCA GCAGG and reverse TCAATTCCCACACACCTCCC; rat CGRP, forward AACCTTAGAA AGCAGCCCAGGCATG and reverse GTGGGCACAAAGTTGTCCTTCACCA; rat actin, forward AGCCATGTACGTAGCCATCC and reverse GCCATCTCTTGCTCGAAGTC. Multiplex PCR amplification was performed in a 20-μl reactive system containing: a 10 μM concentration of each primer (forward, reverse) 0.2 μl, reverse transcription (RT) product 2 μl, dNTP mix (10 mM each) 0.2 μl, Taq polymerase 0.2 μl (2 U), 10 × PCR buffer (Takara) 10 μl, adjusted to the final volume (20 μl) with ddH_2_O. PCR amplification consisted of 30 cycles of denaturation at 94°C for 30 s, annealing at 57°C for 30 s, and elongation at 72°C for 30 s; followed by elongation at 72°C for 10 min. Two microliter of the first-round PCR was reamplified in a second PCR according to the same program. No signals were detected in the absence of template. PCR products were separated by electrophoresis on a 2% agarose gel containing 1 mg/ml ethidium bromide and visualized in a UV transilluminator (Bio-Rad).

### Statistical analysis

Statistical analyses were performed with GraphPad Prism 5 for Windows (GraphPad Software, Inc, USA). All data were expressed as mean ± S.E.M. Two-tailed unpaired Student's *t*-test was used for the comparison of the mean values between two groups. One-way analysis of variance (ANOVA) followed by Dunnett's multiple comparison test or two-way ANOVA followed by Bonferroni post-hoc test was used for multiple comparison. Differences with *p *< 0.05 were considered statistically significant.

## Abbreviations

AP: action potential; AHP: afterhyperpolarization; AHP_80%_: AHP duration at 80% repolarization; CFA: complete Freund's adjuvant; CGRP: calcitonin gene-related peptide; CT: AP current threshold; DRG: dorsal root ganglion; HK: heat-killed; HT: high-firing threshold; B4: isolectin B4; i.t.: intrathecal; LT: low-firing threshold; MT: medium-firing threshold; PCR: polymerase chain reaction; PTL: paw withdrawal latency; PWT: paw withdrawal threshold; R_in_: membrane input resistance; RMP: resting membrane potential; RT: reverse transcription; TP: threshold potential; TRPV1: transient receptor potential vanilloid 1; TTX: tetrodotoxin; WDR: wide dynamic range

## Competing interests

The authors declare that they have no competing interests.

## Authors' contributions

QZ participated in the design of the study, carried out all the experiments, and performed the statistical analysis. DF participated in behavior tests and single-cell reverse-transcriptase PCR analysis. JC participated in the data analysis and interpretation. YW and JSH provided scientific advice for experimental design and manuscript preparation. GGX conceived the project, participated in experimental design and interpretation, and wrote the manuscript. All authors have read and approved the final manuscript.

## Supplementary Material

Additional file 1 **Figure S1**. Comparasion of intrinsic membrane properties of small-sized DRG neurons in MRMT-1-rats with or without allodynia and hyperalgesia. (A): Resting membrane potentials (RMP) and action potential threshold (TP). Representative of action potentials evoked by 100-ms, 25-pA depolarizing current pulse, recorded from low-firing threshold DRG neurons. RMP and TP are showed with arrow in the figure. (B): Representative of the AHP amplitude and duration of action potentials evoked by 100-ms, 40-pA depolarizing current pulse, recorded from low-firing threshold DRG neurons. The vertical dot line to trough shows the AHP amplitude, and the horizontal dot line shows the AHP_80%_. Note that action potentials have been amputated due to high gain. (C): Representative of the firing frequency evoked by a 1-s, 300-pA depolarizing current pulse, recorded from low-firing threshold DRG neurons. (a) in naive rat; (b) in MRMT-1-rat without allodynia; (c) in MRMT-1-rat with allodynia. Click here for file

Additional file 3 **Table S1**. Intrinsic electrogenic properties of small-sized DRG neurons in MRMT-1-rats with or without allodynia. Values are presented as mean ± S.E.M with the number given in parentheses. *p < 0.05, **p < 0.01,***p < 0.001, as compared with no allodynia groups, two-tailed Student's *t*-test. RMP: resting membrane potential; R_in_: membrane input resistance; CT: the depolarized current threshold for evoking the 1^st ^action potential; TP: threshold potential; AHP: afterhyperpolarization; AHP_80% _duration: AHP duration at 80% repolarization (AHP_80%_); AP: action potential. Click here for file

Additional file 4 **Table S2**. Summary of TRPV1 and CGRP mRNA expression in LT (CT < 50 pA), MT (CT 50 ~ 100 pA), and HT (CT > 50 pA) DRG neurons using single-cell reverse-transcriptase PCR. Click here for file

Additional file 2 **Figure S2**. Measured values for an action potential recorded from small-sized DRG neurons. (A): amplitude of action potential; (B): overshot of action potential; (C): duration of action potential; (D): amplitude of afterhyperpolarization (AHP); (E): AHP duration at 80% repolarization (AHP_80%_); (F): duration from resting membrane potential (RMP) to threshold potential (TP); the rise rate of TP = (RMP-TP)/duration from RMP to TP. Click here for file
