# Supplementary material for: Enhanced excitability of small dorsal root ganglion neurons in rats with bone cancer pain
Source: Mol Pain. 2012 Apr 3;8:24. doi: 10.1186/1744-8069-8-24 (PMC3379961; doi:10.1186/1744-8069-8-24)
Supplement: Additional file 3 — Table S1. Intrinsic electrogenic properties of small-sized DRG neurons in MRMT-1-rats with or without allodynia. Values are presented as mean ± S.E.M with the number given in parentheses. *p < 0.05, **p < 0.01,***p < 0.001, as compared with no allodynia groups, two-tailed Student's t-test. RMP: resting membrane potential; Rin: membrane input resistance; CT: the depolarized current threshold for evoking the 1st action potential; TP: threshold potential; AHP: afterhyperpolarization; AHP80% duration: AHP duration at 80% repolarization (AHP80%); AP: action potential. [file 1744-8069-8-24-S3.DOC]

**Additional file 3:**   **Table S1.** Intrinsic electrogenic properties of small-sized DRG neurons in MRMT-1-rats with or without allodynia.

|  |  | **RMP** | **R_in_** | **CT** | **TP** | **TP**  **rise rate** | **AP**  **amplitude** | **AP**  **overshot** | **AP**  **duration** | **AHP**  **amplitude** | **AHP_80%_**  **duration** | **AP frequency** |
| --- | --- | --- | --- | --- | --- | --- | --- | --- | --- | --- | --- | --- |
|  |  | **mV** | **MΩ** | **pA** | **mV** | **v/s** | **mV** | **mV** | **ms** | **mV** | **ms** | **Spikes/s** |
| **CT**  **<50**  **pA** | **Naïve** | **-50.6±0.5**  **(29)** | **654.0±20.1**  **(33)** | **29.8±2.0**  **(29)** | **-27.6±0.8**  **(38)** | **0.8±0.1**  **(30)** | **101.8±1.9**  **(34)** | **55.9±1.4**  **(34)** | **44.6±2.9**  **(30)** | **19.2±0.8**  **(40)** | **17.6±1.1**  **(35)** | **20.5±1.8**  **(30)** |
|  | **No allodynia** | **-50.7±0.5**  **(13)** | **626.9±29.9**  **(11)** | **29.6±2.8**  **(14)** | **-26.3±0.5**  **(14)** | **0.7±0.1**  **(11)** | **106.2±3.2**  **(13)** | **57.7±2.3**  **(13)** | **44.9±2.8**  **(13)** | **17.1±0.7**  **(10)** | **18.6±1.1**  **(11)** | **19.1±2.2**  **(15)** |
|  | **Allodynia** | **-45.6±0.5**  **(52) ^***^** | **546.3±15.3**  **(60) ^***^** | **20.9±1.5**  **(44) ^***^** | **-30.7±0.6**  **(55) ^**^** | **0.9±0.1**  **(45) ^*^** | **94.2±1.9**  **(55) ^**^** | **45.7±1.9**  **(47) ^***^** | **34.3±1.4**  **(54) ^***^** | **14.7±0.7**  **(51) ^***^** | **14.6±0.6**  **(49) ^*^** | **31.4±1.9**  **(32) ^***^** |
| **CT**  **50 ~100 pA** | **Naïve** | **-51.7±1.1**  **(12)** | **593.0±22.3**  **(14)** | **85.0±3.3**  **(13)** | **-20.1±0.7**  **(19)** | **1.9±0.2**  **(11)** | **104.7±3.4**  **(17)** | **55.1±1.9**  **(17)** | **24.5±2.1**  **(15)** | **19.7±1.5**  **(16)** | **10.6±1.4**  **(16)** | **10.5±1.7**  **(15)** |
|  | **No allodynia** | **-52.2±1.4**  **(6)** | **592.0±19.4**  **(5)** | **87.1±4.2**  **(11)** | **-19.9±1.2**  **(6)** | **1.6±0.3**  **(6)** | **103.3±4.4**  **(6)** | **53.8±3.4**  **(6)** | **27.8±3.1**  **(5)** | **16.9±0.8**  **(5)** | **13.5±1.2**  **(5)** | **11.0±1.6**  **(4)** |
|  | **Allodynia** | **-48.6±0.5**  **(16) ^**^** | **486.5±25.8**  **(17) ^**^** | **72.2±4.0**  **(16) ^*^** | **-23.4±1.0**  **(17) ^**^** | **2.3±0.2**  **(14)** | **98.7±2.6**  **(17)** | **46.1±2.9**  **(15) ^*^** | **22.1±1.2**  **(17)** | **14.4±1.3**  **(17) ^*^** | **11.4±1.0**  **(17)** | **19.3±3.2**  **(12) ^*^** |
| **CT**  **>100**  **pA** | **Naïve** | **-54.6±2.1**  **(7)** | **550.5±40.8**  **(9)** | **157.9±12.6**  **(7)** | **-16.6±1.0**  **(8)** | **5.4±1.1**  **(5)** | **103.7±3.6**  **(7)** | **54.1±3.3**  **(5)** | **17.5±3.2**  **(6)** | **10.8±1.3**  **(8)** | **6.3±0.6**  **(6)** | **7.1±3.4**  **(8)** |
|  | **No allodynia** | **-53.0±1.4**  **(5)** | **553.7±80.5**  **(5)** | **184.0±17.2**  **(5)** | **-15.8±0.3**  **(4)** | **4.9±0.5**  **(4)** | **109.1±3.8**  **(5)** | **57.8±3.6**  **(5)** | **15.2±0.7**  **(5)** | **8.6±3.2**  **(5)** | **5.9±1.5**  **(5)** | **6.5±4.3**  **(4)** |
|  | **Allodynia** | **-51.2±0.9**  **(13)** | **508.1±24.9**  **(12)** | **161.7±16.6**  **(9)** | **-20.2±1.4**  **(9)** | **5.0±0.9**  **(7)** | **103.3±4.9**  **(10)** | **54.9±2.6**  **(8)** | **13.9±1.4**  **(9)** | **9.0±1.39**  **(10)** | **6.3±0.9**  **(7)** | **10.0±4.3**  **(8)** |

Values are presented as mean ± S.E.M with the number given in parentheses. ^*^*p*<0.05, ^**^*p*<0.01,^***^*p*<0.001, as compared with no allodynia groups, two-tailed Student’s *t*-test. RMP: resting membrane potential; R_in_: membrane input resistance; CT: the depolarized current threshold for evoking the 1^st^ action potential; TP: threshold potential; AHP: afterhyperpolarization; AHP_80%_ duration: AHP duration at 80% repolarization (AHP_80%_); AP: action potential.
