# Supplementary material for: Enhanced excitability of small dorsal root ganglion neurons in rats with bone cancer pain
Source: Mol Pain. 2012 Apr 3;8:24. doi: 10.1186/1744-8069-8-24 (PMC3379961; doi:10.1186/1744-8069-8-24)
Supplement: Additional file 4 — Table S2. Summary of TRPV1 and CGRP mRNA expression in LT (CT < 50 pA), MT (CT 50 ~ 100 pA), and HT (CT > 50 pA) DRG neurons using single-cell reverse-transcriptase PCR. [file 1744-8069-8-24-S4.DOC]

**Additional file 4:**  **Table S2.** Summary of TRPV1 and CGRP mRNA expression in LT (CT < 50 pA), MT (CT 50 ~ 100 pA), and HT (CT > 50 pA) DRG neurons using single-cell reverse-transcriptase PCR.

|  | **TRPV1^+^/CGRP^+^** | **TRPV1^+^/CGRP^-^** | **TRPV1^-^/CGRP^+^** | **TRPV1^-^/CGRP^-^** | **Total** |
| --- | --- | --- | --- | --- | --- |
| **CT**  **<50 pA** | **12**  **(34.3%)** | **16**  **(45.7%)** | **4**  **(11.4%)** | **3**  **(8.6%)** | **35** |
| **CT**  **50~100 pA** | **2**  **(18.2%)** | **6**  **(54.5%)** | **1**  **(9.1%)** | **2**  **(18.2%)** | **11** |
| **CT**  **>100 pA** | **1**  **(10%)** | **5**  **(50%)** | **2**  **(20%)** | **2**  **(20%)** | **10** |

| **TRPV1^+^** | **CGRP^+^** | **TRPV1-/CGRP-** | **Total** |
| --- | --- | --- | --- |
| **42 (75.0%)** | **22 (39.3%)** | **7 (12.5%)** | **56** |
