# Supplementary figures and images for: Enhanced excitability of small dorsal root ganglion neurons in rats with bone cancer pain
Source: Mol Pain. 2012 Apr 3;8:24. doi: 10.1186/1744-8069-8-24 (PMC3379961; doi:10.1186/1744-8069-8-24)

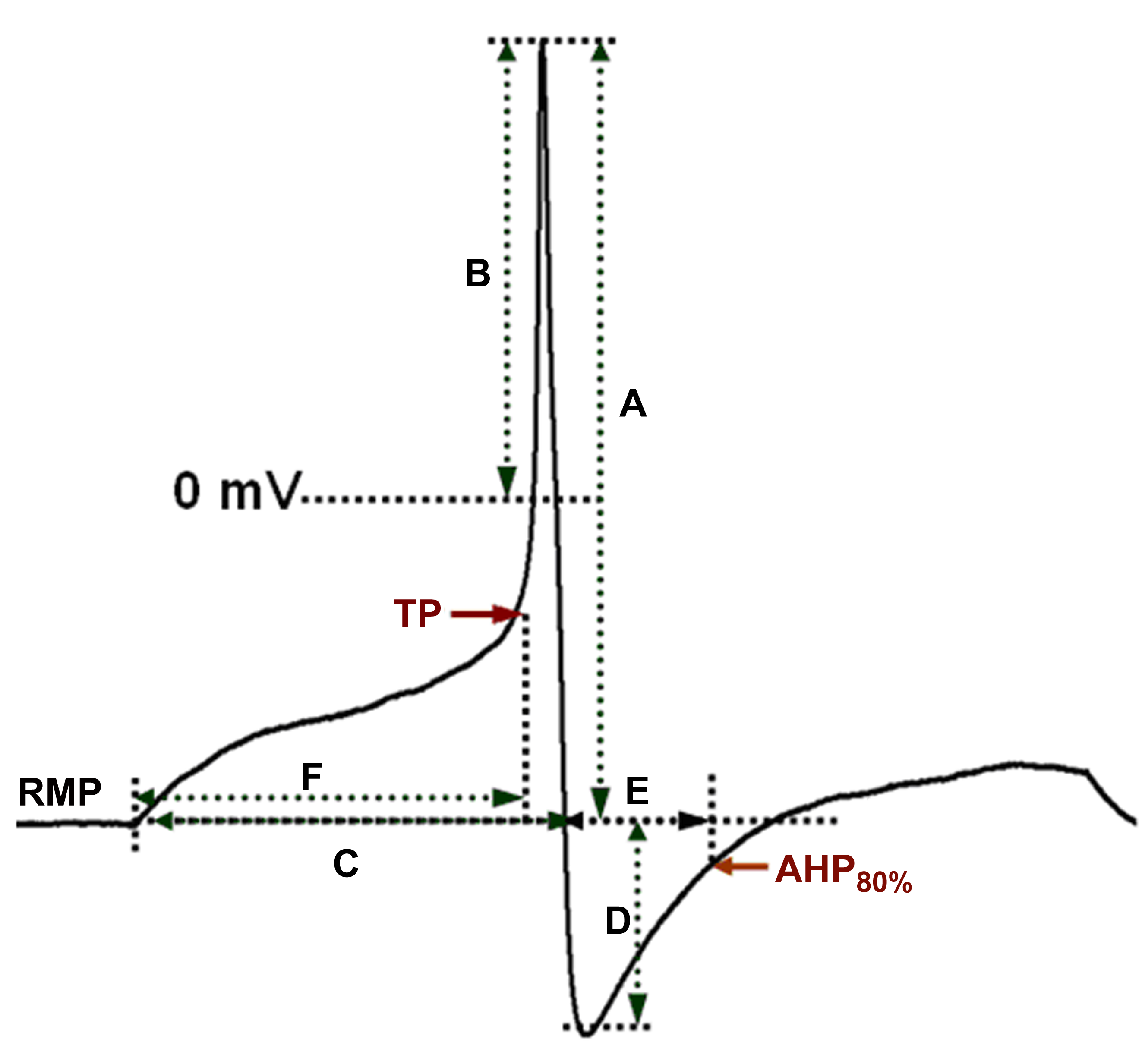

Supplement: Additional file 2 — Figure S2. Measured values for an action potential recorded from small-sized DRG neurons. (A): amplitude of action potential; (B): overshot of action potential; (C): duration of action potential; (D): amplitude of afterhyperpolarization (AHP); (E): AHP duration at 80% repolarization (AHP80%); (F): duration from resting membrane potential (RMP) to threshold potential (TP); the rise rate of TP = (RMP-TP)/duration from RMP to TP. [file 1744-8069-8-24-S2.TIFF]
